# CRISPR-Cas9-based electrochemical biosensor for the detection of *katG* gene mutations in isoniazid-resistant tuberculosis

**DOI:** 10.5599/admet.2766

**Published:** 2025-06-17

**Authors:** Dika Apriliana Wulandari, Muhammad Ihda Hamlu Liwaissunati Zein, Salma Nur Zakiyyah, Safri Ishmayana, Mehmet Ozsoz, Yeni Wahyuni Hartati, Irkham

**Affiliations:** 1 Department of Chemistry, Faculty of Mathematics and Natural Science, Universitas Padjadjaran, Sumedang 45363, Indonesia; 2 Department of Chemistry “Giacomo Ciamician”, Alma Mater Studiorum - University of Bologna, Bologna 40126, Italy; 3 Department of Biomedical Engineering, Near East University, Mersin 99138, Turkey; 4 Study Center of Sensor and Green Chemistry, Faculty of Mathematics and Natural Science, Universitas Padjadjaran, Bandung 40132, Indonesia

**Keywords:** Electrochemistry, guide RNA, ferrocene signal, drug-resistant tuberculosis

## Abstract

**Background and purpose:**

Multidrug-resistant tuberculosis (MDR-TB) remains a significant challenge in tuberculosis (TB) treatment, driven by simultaneous mutations in the *rpoB* and *katG* genes that confer resistance to rifampicin and isoniazid. While many molecular diagnostic tools focus on *rpoB*, the *katG* gene is often overlooked despite its critical role in confirming MDR-TB. This study aims to develop a CRISPR/Cas9-based electrochemical biosensor for the rapid and selective detection of *katG* mutation.

**Experimental approach:**

A guide RNA (gRNA) specific to the mutation site on *katG* gene was designed using the Benchling CRISPR tool, considering on-target and off-target scores, specificity, and cleavage sites within the *Mycobacterium tuberculosis* genome. The selected gRNA achieved the highest on-target score of 61.2 and an off-target score of 49.0 at cut position 2928, with a PAM sequence of AGG. Its cleavage efficiency was validated experimentally using an electrochemical biosensing platform incorporating a gold-modified screen-printed carbon electrode (SPCE/Au). Redox response enhancement by [Fe(CN_6_)]^3-/4-^ confirmed the improved performance of the electrode.

**Key results:**

The biosensor system detects the target DNA through hybridization with DNA probe-Fc, forming double-stranded DNA (dsDNA) that is recognized and cleaved by the Cas9/gRNA complex. This cleavage significantly reduces the ferrocene oxidation signal, indicating the presence of a *katG* mutation. Non-mutated target DNA produces a nondetectable ferrocene signal, suggesting that the Cas9 enzyme may remain bound to the electrode without cleavage. The CRISPR/Cas9 electrochemical biosensor demonstrated a low detection limit of 7.5530 aM and a detection range of 10^1^ to 10^6^ aM.

**Conclusion:**

The CRISPR/Cas9-based electrochemical biosensor exhibits high sensitivity and specificity for the detection *katG* mutation, offering a promising platform for rapid MDR-TB diagnostics.

## Introduction

Tuberculosis (TB) remains the second leading cause of infectious disease-related mortality worldwide, responsible for nearly twice as many deaths as HIV/AIDS. Over 10 million people are affected annually, and since 2021, TB incidence has continued to rise [1]. TB is caused by the aerobic bacterium *Mycobacterium tuberculosis* (MTB), which commonly infects oxygen-rich tissues [2,3]. The emergence of drug-resistant TB strains, particularly multidrug-resistant TB (MDR-TB), poses a significant challenge to global TB control efforts [4-6]. These strains often harbour genetic mutations that allow them to evade the effects of commonly used drugs [7].

One of the most critical drugs in TB treatment is isoniazid (INH). Resistance to INH, often due to mutations in the *katG* gene, is a major contributor to treatment failure [8-11]. Traditional diagnostic methods, such as bacterial culture, are time-consuming and lack sensitivity and specificity [12,13]. In 2010, the World Health Organization (WHO) recommended the use of GeneXpert as a diagnostic tool for early detection of MDR-TB, replacing traditional smear microscopy [8]. While GeneXpert is effective in detecting rifampicin resistance (RIF-R), it cannot identify resistance to isoniazid (INH), a key drug in TB treatment [14]. Therefore, there is a pressing need for rapid, sensitive, and specific diagnostic tools to detect *katG* mutations associated with INH resistance, enabling more accurate MDR-TB diagnosis and effective patient management.

CRISPR/Cas9-based molecular diagnostics have emerged as promising tools for rapid and precise detection of nucleic acid mutations and for point-of-care (POC) applications [15-18]. The technology is also highly sensitive, enabling the detection of low levels of nucleic acids, and provides rapid results, allowing for timely diagnosis of infectious diseases or genetic disorders [19-21]. The CRISPR system consists of two main components: the Cas protein, which acts as an endonuclease, and the guide RNA (gRNA), which directs Cas to the specific sequence. The Cas protein and gRNA combine to form a ribonucleoprotein (RNP) complex, which becomes active when it encounters target DNA complementary to the gRNA and recognized by the protospacer adjacent motif (PAM) sequence. The cutting efficiency of the CRISPR/Cas system is influenced by factors including the design of the gRNA specific to the target DNA sequence [22]. The single-guide RNA (sgRNA) is a modified version of the naturally occurring two-part gRNA complex, combined into an RNA sequence. It contains the crRNA, which directs Cas9 to the target site, and the tracrRNA, which forms a scaffold for Cas9 binding. Precise targeting is achieved by synthesizing an RNA molecule that consists of a gRNA complementary to the target strand, along with a constant tracrRNA [23,24]. An essential characteristic of the CRISPR/Cas system is the PAM, a conserved DNA sequence adjacent to the target site that is CRISPR-dependent [25].

Recent advancements have integrated CRISPR/Cas9 with electrochemical biosensors to create sensitive, specific, and rapid platforms for diagnostics. When combined with the precision targeting capability of CRISPR/Cas9, electrochemical biosensors can be transformed into programmable platforms capable of detecting specific genetic mutations. The CRISPR/Cas9 system can be programmed with a guide RNA (gRNA) to recognize a precise DNA sequence adjacent to a protospacer adjacent motif (PAM), resulting in a site-specific cleavage event. In a biosensor context, this cleavage can be transduced into an electrochemical signal via redox-active labels (*e.g.* ferrocene, methylene blue) or changes in conductivity at the electrode interface [26-28].

**Figure 1.**
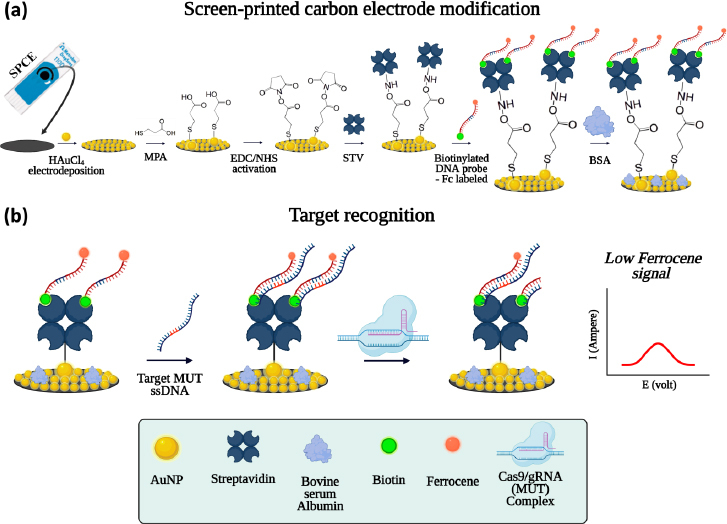
Biosensor mechanism of **(a)** SPCE modification, **(b)** target recognition of CRISPR/Cas9-based electrochemical biosensor for the detection of *katG* gene mutations

In this study, we developed a CRISPR/Cas9-based electrochemical biosensor for the specific detection of *katG* gene mutations associated with INH resistance. A mutation-specific gRNA was designed using the Benchling CRISPR tool and applied in a biosensor platform incorporating a ferrocene-labelled mutant DNA probe. The biosensor employed a two-step mechanism (Figure 1), screen-printed carbon electrodes (SPCE) were modified with gold nanoparticles (AuNPs) and functionalized via streptavidin-biotin interaction to immobilize the ferrocene-labelled probe. The complementary mutant DNA target hybridized with the probe and was cleaved explicitly by the Cas9/gRNA complex. The cleavage event disrupted the ferrocene signal, generating a measurable change in current. This system offers a promising approach for rapid, accurate, and field-deployable TB diagnostics.

## Experimental

### Materials and reagents

The materials used in this research were aqua pro injection (PT Ikapharmindo Putramas), bovine serum albumin (BSA; Sigma Aldrich), chloroauric acid (HAuCl_4_ .3H_2_O; Sigma Aldrich), DEPC treated water (Nippon Gene, Japan), DNA probe-Ferrocene (5’Biotin-TTCCTGCGCTAGTGGTGGCCGTAGCTCCAGCAT-Fc3’; Genescript, Singapore), DNA target mutant (5’CGGAACCGGTAAGGACGCGATCACCACCGGCATCGAGGTCGTATGGACG3’; Facmac, Japan), DNA target wild type (5’CGGAACCGGTAAGGACGCGATCACCAGCGGCATCGAGGTCGTATGGACG3’; Facmac, Japan), sgRNA (5’CGCGAUCACCACCGGCAUCG; Genescript, Singapore), ethanol absolute (C_2_H_6_O; Merck), 1-(3-dimethylaminopropyl)-3-ethyl carbodiimide hydrochloride (EDC; Sigma Aldrich), 3-mercaptopropionic acid (MPA; Sigma Aldrich), N-hydroxysuccinimide (NHS; Sigma Aldrich), potassium chloride (KCl; Merck), potassium ferricyanide ([K_3_[Fe(CN)_6_]; Sigma Aldrich), phosphate buffer saline 10x (PBS; HiMedia, India), streptavidin (Promega), sulfuric acid (H_2_SO_4_; Merck), and Screen-printed carbon electrode consists of carbon-based working (diameter of 0.40 cm), carbon auxiliary electrodes, and silver as a reference electrode (Metrohm DropSens, Spain).

### Instrumentation

The instrument used was ZP potentiostat, which was connected to a computer using PSTrace 5.11 software (Zimmer & Peacock, UK), a scanning electron microscope (SEM, JEOL JCM-6000), an incubator (JoanLab), a micropipette (DragonLab), a microtube (Labselect), and a micropipette tip.

### DNA sequence database of MTB

The gRNA sequence was determined from the whole genome sequence of MTB (H37Rv) that is available at the Genbank National Center of Biotechnology Information (NCBI) [29], with the sequences of the *rpoB* gene [30] and *katG* gene [31].

### Prediction of CRISPR/Cas9 gRNA sequences

The selection of gRNA for detecting mutations in INH-R involves identifying the mutation points at specific amino acid positions, as determined from studies analysing the distribution of mutations across various regions. Based on mutation distribution data for the *katG* genes isolates from various geographic regions (Supplementary Table S1 and Supplementary Table S2), it can be concluded that the most frequent mutations in the *katG* gene are predominantly located at amino acid position 315. Therefore, the selection of gRNA candidates can be guided by the mutation patterns in the *katG* genes across these regions.

Candidate gRNAs were identified using the Benchling platform by examining the highest on-target and off-target scores. Additionally, the determination of gRNA candidates for mutated DNA by modifying the side of the mutations in wild-type gRNA.

### Modification of SPCE with gold nanoparticles

SPCE was modified with AuNPs using the electrodeposition method. The electrode was first treated with deionized water and dried at room temperature. Pre-treatment was carried out in 0.1 M H_2_SO_4_ using cyclic voltammetry for 10 cycles. Then, 50 μL of 1.0 mM HAuCl_4_ in 0.1 M KCl was dropped onto the electrode surface and electrodeposited using chronoamperometry. After modification, the SPCE was rinsed with distilled water and dried. The modified electrode was characterized using differential pulse voltammetry (DPV) in 10 mM K_3_[Fe(CN)_6_] with 0.1 M KCl (conditions: pretreatment settings: *E* condition: -0.2 V; *t* condition: 10 s; DPV settings: *t*_equilibration_: 0 s, *E*_begin_: -0.2 V, *E*_end_: 0.5 V; *E*_step_: 0.004 V; *E*_pulse_: 0.025 V; *t*_pulse_: 0.05 s, scan rate 0.008 V s-1) to observe the redox response of the modified surface.

### Immobilization of ferrocene-labelled DNA probe (DNA probe-Fc)

The SPCE/AuNPs electrode was functionalized with 0.01 M MPA and incubated at 25 °C for 20 minutes, followed by washing with an ethanol-water mixture (1:3). Activation was performed by adding 0.1 M EDC/NHS (1:1), incubating at 25 °C for 60 minutes, and rinsing with distilled water. Then, 3 μL of streptavidin (50 ppm) was added to the surface and incubated at 4 °C for 90 minutes, followed by washing with 1× PBS. Subsequently, 3 μL of DNA probe-Fc (2 μM) was immobilized onto the surface and incubated at 25 °C for 60 minutes. After washing with 1× PBS, 3 μL of 0.01 % BSA was added and incubated at 25°C for 10 minutes to block non-specific sites. The final electrode was rinsed again with 1× PBS. Electrochemical characterization was performed using square wave voltammetry (SWV) in 1× PBS (conditions: equilibration time 0 s, potential range -0.1 to 0.5 V, step potential 0.004 V, amplitude 0.025 V, and frequency 25 Hz) to measure the redox signal of the ferrocene label.

### Target recognition and cleavage by the Cas9-gRNA complex

A mixture of gRNA (100 nM), Cas9 (100 nM), RNase inhibitor, 10× Cas9 reaction buffer, and 1× PBS was prepared in a microtube and incubated at 37 °C for 10 minutes to form the Cas9-sgRNA complex. Separately, the target single-stranded DNA (ssDNA) was applied onto the SPCE/AuNPs/MPA-EDC-NHS/STV/DNA probe-Fc-modified electrode and incubated at 37 °C for 10 minutes, followed by washing with 1× PBS. The Cas9-sgRNA complex was added to the electrode surface and incubated at 37 °C for 120 minutes. After incubation, the electrode was rinsed with 1× PBS. Electrochemical characterization was performed using SWV in 1× PBS (conditions: equilibration time 0 s, potential range -0.1 to 0.5 V, step potential 0.004 V, amplitude 0.025 V, and frequency 25 Hz). The resulting ferrocene redox current indicated DNA cleavage activity, with lower current responses observed in the presence of target mutations compared to wild-type or non-target sequences.

## Results and discussion

### gRNA design for CRISPR/Cas9 target prediction

The on-target and off-target scores are critical metrics for identifying optimal CRISPR/Cas9 gRNA candidates. The on-target score measures the binding efficiency of the gRNA to the target DNA, ensuring accurate cleavage by the Cas enzyme. A higher on-target score indicates greater precision and effectiveness of the gRNA in directing Cas9 to the intended target. In contrast, the off-target score quantifies the likelihood of the gRNA binding to and facilitating cleavage of non-target DNA. A higher off-target score reflects a reduced probability of unintended interactions, minimizing off-target effects [32].

The algorithms developed by Doench *et al.* [33] and Hsu *et al.* [34] were employed to calculate the on-target and off-target scores. For on-target score calculation, factors such as the position and length of the gRNA sequence, the PAM sequence specific to the Cas9 enzyme (NGG for SpCas9, N represents any of the four nucleotide bases), and the secondary structure formation energy (*e.g.* hairpins) in the gRNA that can affect Cas9 binding and cleavage efficiency are considered [33]. Conversely, the off-target score is determined by factors including the presence of DNA sequences in the genome similar to the target sequence particularly those near the PAM the position and number of mismatches between the gRNA and target DNA, and the location of the PAM, as sequences proximal to the PAM are more prone to off-target cleavage [34].

The gRNA designs targeting the *katG* gene at amino acid codons 315 are presented in Table 1. This table illustrates that codon 315 is associated with a specific gRNA design, exhibiting various values for on-target and off-target scores.

**Table 1.** gRNA design targeting *katG* gene at amino acid codon position 315 (wild-type)

Cut position	Strand	Guide sequence (5’ - 3’)	PAM	On-target score	Off-target score
2920	+	GGTAAGGACGCGATCACCAG	CGG	68.9	49.4
2928	+	CGCGATCACCAGCGGCATCG	AGG	61.2	49.0
2925	-	CCATACGACCTCGATGCCGC	TGG	57.9	50.0
2936	+	CCAGCGGCATCGAGGTCGTA	TGG	50.3	49.9
2907	-	GCTGGTGATCGCGTCCTTAC	CGG	36.6	49.9

In designing gRNA for the CRISPR/Cas system, the relationship between the location of the mutation points and the PAM sequence is critical because the PAM is essential for target recognition by the Cas enzyme. If the mutation point is located too far from the PAM (e.g. more than 20-25 base pairs), the gRNA may not effectively direct the Cas enzyme to the target because the target sequence must be within the 20 nucleotides preceding the PAM. Mutations located close to the PAM sequence can still be targeted by CRISPR/Cas, provided the PAM remains intact and the gRNA is designed to account for the changes in the target sequence [35,36].

In CRISPR-based mutation detection, the gRNA design should utilize specific differences between normal and mutant targets. The mutation should be located near the 3' end of the gRNA target sequence (before the PAM), as this region is more sensitive to the recognition of mismatched base pairs, allowing CRISPR to more effectively distinguish between normal and mutated targets. If the mutation is too far from the PAM or located at the 5' end of the target sequence, the sensitivity to mismatches tends to decrease, which may compromise the specificity of detection [36].

The relationship between mutation distance and PAM sequence in CRISPR/Cas9-based sensors has been studied in previous research, where it was explained that the closer the distance of the mutation point to the PAM, the lower the sensor signal and almost the same as the signal without the target. On the other hand, the response will be nearly identical to the full match target signal the farther the mutation site is from the sensor. The optimal mutation and PAM distance that results in a distinct difference between the no-target and target-present signals is at a distance of 10 bases [37,38].

**Figure 2.**
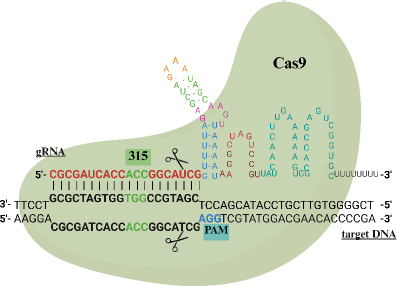
The illustration of gRNA targeting *katG* gene at codon 315 with the PAM sequence AGG

Mutations in the *katG* gene can be identified by designing gRNA specifically targeting the mutation site. The mutation-specific adjusted gRNA designs for the *katG* gene are provided in Table 2. In biosensor applications, the distance between the mutation site and the protospacer adjacent motif (PAM) sequence is a critical factor in gRNA design. Accordingly, the selected gRNA targets codon 315 of the *katG* gene, which exhibits high on-target and off-target scores of 61.2 and 49.0, respectively. The most common mutation at this codon involves a nucleotide substitution from AGC to ACC, located 7 bases from the PAM site. Figure 2 presents the gRNA candidate mechanism for the *katG* gene, targeting the cleavage site at position 2928 with an AGG recognition sequence.

**Table 2.** gRNA design targeting *katG* gene frequent mutation at amino acid codon position 315

Nucleotide change	Amino acid change	Cut position	Strand	Guide sequence mutation (5’ - 3’)	PAM	On-target score	Off-target score	Mutation distance with PAM
AGC 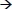 ACC	Ser 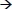 Thr	2920	+	GGTAAGGACGCGATCACCAC	CGG	68.9	49.4	1 base
AGC 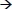 AAC	Ser 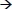 Asn			GGTAAGGACGCGATCACCAA				1 base
AGC 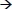 ATC	Ser 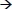 Ile			GGTAAGGACGCGATCACCAT				1base
AGC 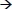 AGA	Ser 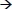 Arg			-				-
AGC 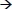 ACC	Ser 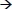 Thr	2928	+	CGCGATCACCACCGGCATCG	AGG	61.2	49.0	8 bases
AGC 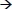 AAC	Ser 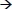 Asn			CGCGATCACCAACGGCATCG				8 bases
AGC 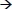 ATC	Ser 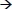 Ile			CGCGATCACCATCGGCATCG				8 bases
AGC 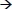 AGA	Ser 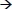 Arg			CGCGATCACCAGAGGCATCG				7 bases
AGC 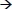 ACC	Ser 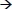 Thr	2925	-	CCATACGACCTCGATGCCGG	TGG	57.9	50.0	16 bases
AGC 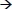 AAC	Ser 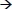 Asn			CCATACGACCTCGATGCCGT				16 bases
AGC 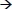 ATC	Ser 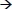 Ile			CCATACGACCTCGATGCCGA				16 bases
AGC 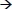 AGA	Ser 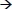 Arg			CCATACGACCTCGATGCCTC				15 bases
AGC 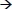 ACC	Ser 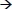 Thr	2936	+	CCACCGGCATCGAGGTCGTA	TGG	50.3	49.9	16 bases
AGC 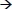 AAC	Ser 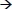 Asn			CCAACGGCATCGAGGTCGTA				16 bases
AGC 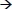 ATC	Ser 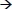 Ile			CCATCGGCATCGAGGTCGTA				16 bases
AGC 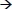 AGA	Ser 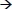 Arg			CCAGAGGCATCGAGGTCGTA				15 bases
AGC 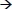 ACC	Ser 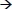 Thr	2907	-	GGTGGTGATCGCGTCCTTAC	CGG	36.6	49.9	1 base
AGC 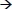 AAC	Ser 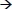 Asn			GTTGGTGATCGCGTCCTTAC				1 base
AGC 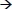 ATC	Ser 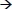 Ile			GATGGTGATCGCGTCCTTAC				1 base
AGC 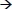 AGA	Ser 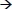 Arg			TCTGGTGATCGCGTCCTTAC				0 bases

To verify the specificity and effectiveness of the selected gRNA against the mutant target DNA, an electrochemical biosensor assay was conducted. This test aimed to confirm that the gRNA can selectively bind and induce cleavage at the mutated DNA site, and to prove that this concept, combined with an electrochemical biosensor system, could detect MDR-TB quantitatively and could differentiate the signal from the non-mutated TB. Additionally, the electrochemical biosensor enabled real-time monitoring of the gRNA-target interaction, providing valuable insights into the efficiency of the Cas9-mediated DNA cutting process. The integration of this approach offers a rapid, sensitive, and reliable method for assessing gRNA performance, which could have significant implications for diagnostic applications.

### SPCE modification with AuNPs

SPCE were pretreated with sulfuric acid (H_2_SO_4_) using cyclic voltammetry (CV) to remove organic binders and surface contaminants, introduce oxygen-containing functional groups (*e.g.* hydroxyl and carbonyl), and increase the electroactive surface area to improve the electrochemical performance of the electrode [39]. Following the pretreatment process, the SPCE were modified with AuNPs via electrochemical deposition. his method offers precise control over key parameters, including deposition time, applied potential, and metal ion concentration. In this study, electrodeposition was performed using the chronoamperometry (CA) technique, which applies a constant potential for a set duration while measuring the associated current response [40]. During this process, tetrachloroaurate ions (AuCl_4_^–^) undergo electrochemical reduction to solid gold (Au^0^), which deposits onto the electrode surface. A potential of -0.1 V was applied to facilitate the formation of AuNPs, selected based on the reduction peak of HAuCl_4_ to Au^0^ [41].

**Figure 3.**
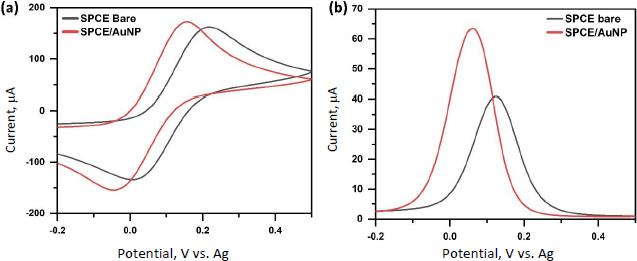
(a) cyclic voltammogram (b) differential pulse voltammogram, of SPCE bare and SPCE/AuNPs using a redox system of 10 mM K_4_[Fe(CN)_6_] in a 100 mM KCl solution

The electrochemical performance of the SPCE modified AuNPs was evaluated using CV and DPV in the presence of the standard redox probe [Fe(CN)_6_]^3-/4-^. The CV results showed a decrease in ΔE from 0.2449 V for the bare SPCE to 0.2023 V for SPCE/Au, indicating improved electron transfer kinetics after AuNPs modification (Figure 3a). This enhancement is further supported by DPV measurements, which revealed a 1.5-fold increase in peak current compared to the bare SPCE, confirming that AuNPs significantly facilitate electron transfer at the electrode surface (Figure 3b). The increased peak current can be attributed to the high surface area and higher conductivity of AuNPs, which provide more active sites and promote faster electron exchange between the redox probe and the electrode. Overall, these results demonstrate that the modification with AuNPs effectively improves the electrochemical properties of the electrode.

### DNA probe immobilization via the streptavidin-biotin system

The designed DNA probe incorporates a biotin group at the 3′ end and a ferrocene label at the 5′ end. The biotin-end facilitates immobilization onto sensor surfaces via the streptavidin-biotin interaction. The streptavidin-biotin system is extensively employed in biosensor applications due to its exceptionally strong and specific non-covalent interaction, characterized by a dissociation constant (*K*_d_ ≈ 10^-14^ M). This high-affinity binding facilitates the stable and efficient immobilization of biotinylated biomolecules, enhancing the sensitivity and reliability of biosensors [42-44]. The integration of 3-mercaptopropionic acid (MPA) with EDC/NHS chemistry is a prevalent method for the covalent immobilization of biomolecules on biosensor surfaces, particularly gold electrodes. MPA forms a self-assembled monolayer (SAM) on gold, exposing carboxyl groups that can be activated by EDC (1-ethyl-3-(3-dimethylaminopropyl)carbodiimide) and NHS (N-hydroxysuccinimide) to generate reactive esters. In this experiment, these esters readily react with primary amine groups of biomolecules, such as streptavidin, forming a stable amide bond that attaches the biomolecules onto the sensor surface [45].

After the biotinylated DNA probe-Fc is immobilized onto the SPCE surface via streptavidin-biotin binding, BSA is immobilized as a blocking agent. BSA effectively occupies unbound sites on the sensor surface, thereby minimizing nonspecific adsorption of biomolecules. This reduction in nonspecific binding decreases background noise and enhances the sensitivity of the biosensor assay [46].

**Figure 4.**
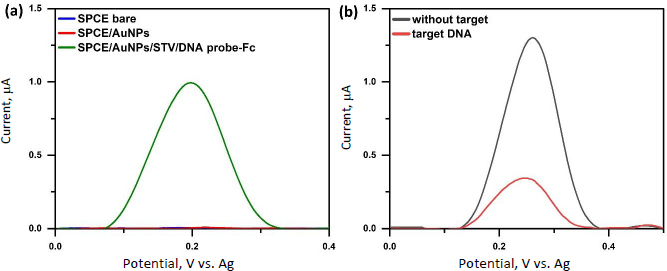
Square wave voltammogram characterization of ferrocene redox system (a) at SPCE bare, SPCE/AuNPs, SPCE/AuNPs/STV/DNA probe-Fc (b) for target recognition using CRISPR/Cas9 system, in 1x PBS solution

**Figure 5.**
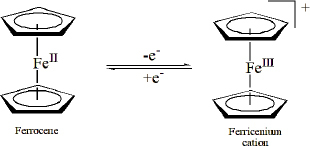
Ferrocene/Ferrocene^+^ redox reaction, created by the author based on information from [47]

The modification of the DNA probe on the SPCE and the application of the CRISPR/Cas9 system for target DNA mutation recognition were characterized using SWV (Figure 4). A ferrocene peak was observed on SPCE/AuNPs/STV/DNA probe-Fc (Figure 4a). Ferrocene is an electroactive molecule that undergoes a reversible electron oxidation to form ferrocenium at a potential of approximately +0.2 V, as represented by the redox reaction in Figure 5. This peak and redox behaviour confirms the successful immobilization of the ferrocene-labelled DNA probe on the electrode surface and its electrochemical activity.

The hybridization of the complementary target DNA with the ferrocene-labelled probe DNA on the surface of the SPCE facilitates the formation of double-stranded DNA (dsDNA), which is crucial for recognition and cleavage by the Cas9/gRNA complex. Cas9, guided by the sgRNA, specifically binds to the protospacer adjacent motif (PAM) sequence and induces local DNA strand separation, enabling the formation of an R-loop structure necessary for targeted dsDNA cleavage [48]. Electrochemical characterization using SWV revealed a significant decrease in the ferrocene oxidation current in the presence of the target DNA compared to the blank (without target), as shown in Figure 4b. This reduction in current indicates successful cleavage of the dsDNA by the Cas9/gRNA complex, resulting in the release of ferrocene-labelled DNA fragments from the electrode surface, which decreases the electrochemical signal.

### Electrochemical performance of the CRISPR/Cas9-based biosensor

**Figure 6.**
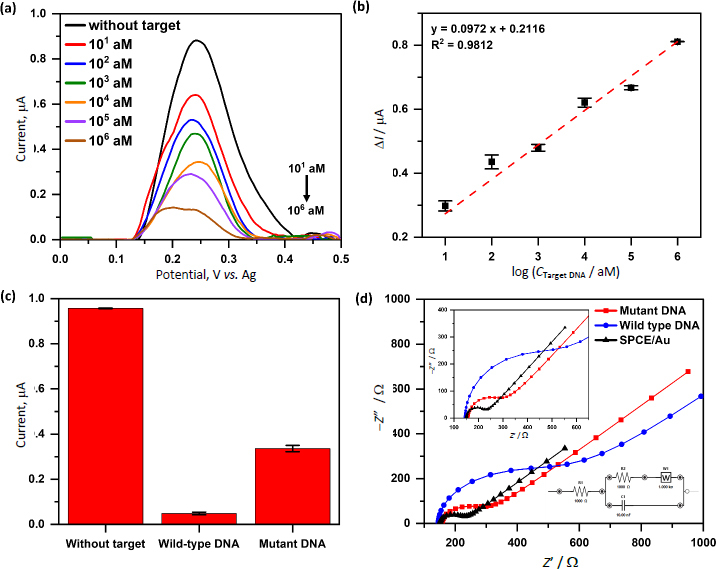
(a) Square wave voltammogram characterization of ferrocene redox system at various concentrations (10^1^, 10^2^, 10^3^, 10^4^, 10^5^, 10^6^ aM) of target DNA mutant in in 1x PBS solution. (b) Plot of Δ*I vs.* log concentration of target DNA mutant in Figure 5a. The error bar represents the measurement precision with *n* = 2. (c) Histogram comparing current responses of the CRISPR/Cas9-based biosensor for without, wild-type, and mutant DNA target. (d) Nyquist plots of electrochemical impedance spectroscopy for SPCE/Au (black), wild-type (blue), and mutant (red) DNA target

In this study, different concentrations of mutant target DNA (from 10^1^ to 10^6^ aM) were tested to evaluate the response trend of the CRISPR-based electrochemical biosensor (Figures 6a and 6b). The voltammogram showed a decrease in ferrocene signal as DNA concentration increased, indicating more DNA cleavage by the Cas9 enzyme. The current difference (Δ*I*) between each sample and the blank was used to construct a calibration curve, yielding a regression equation of *y* = 0.0972*x* + 0.2116 with a strong correlation (*R*^2^ = 0.9812). The detection limit was calculated to be 7.5530 aM, with a linear response range from 10^1^ to 10^6^ aM. The results in Figure 6a were obtained using different electrodes for each concentration to assess inter-electrode reproducibility. Relative standard deviation (RSD) values varied depending on the concentration, ranging from a maximum of 5.27 % at 10^1^ aM to a minimum of 0.05 % at 10^6^ aM, indicating high precision and consistent sensor performance across the tested range. These findings confirm the biosensor’s capability to detect very low levels of mutated DNA, which is crucial for the early detection of drug-resistant tuberculosis.

Furthermore, the CRISPR/Cas9-based electrochemical biosensor developed in this study was tested towards non-mutated, wild-type TB DNA targets, which contained a single mismatch at position 8 from the PAM site (Figure 6c). The mutant target (fully complementary to the gRNA) was cleaved by Cas9, resulting in a decreased ferrocene current due to the disruption of probe conformation and electron transfer. However, the wild-type target shows a complete loss of ferrocene current. This phenomenon is likely due to the fact that Cas9 can still bind stably to DNA targets containing single mismatches in the seed region (positions 1-8 from the PAM site), without inducing cleavage. A previous study utilized Förster Resonance Energy Transfer (FRET) to show that such mismatches prevent the HNH nuclease domain from adopting the active conformation necessary for DNA cleavage, yet the Cas9/gRNA complex remains bound to the DNA [49,50]. This persistent binding forms a substantial complex on the electrode surface, potentially blocking the ferrocene oxidation completely, obstructing electron transfer from the ferrocene label to the electrode, resulting in the observed loss of redox current. Although cleavage is inhibited, the Cas9/gRNA/DNA complex remains intact. This large complex (/160 kDa), when formed on the sensor surface, likely introduces significant steric hindrance that physically blocks electron transfer between the ferrocene moiety and the electrode.

To further investigate this phenomenon, electrochemical impedance spectroscopy (EIS) was employed to characterize changes in interfacial charge transfer resistance (*R_c_*_t_) at the modified electrode surface (Figure 6d). The results revealed a significantly higher *R*_ct_ value (355.1 ) in the wild-type target compared to the mutant target. This increase in *R*_ct_ confirms that the surface becomes more resistive upon wild-type DNA binding, consistent with the formation of a large, non-cleaved Cas9/gRNA/DNA complex that physically blocks access of the ferrocene moiety to the electrode surface. In contrast, the mutant target, which undergoes cleavage, showed lower *R*_ct_ values (123.7 Ω) due to the release of the ferrocene-labelled DNA fragment, which restores electron transfer for the [Fe(CN)_6_]^3-/4-^ and allows partial redox current to be detected. This result confirms that ferrocene signal attenuation in the wild type is not due to cleavage, but due to steric blockade by the Cas9 complex, which remains bound to the DNA. This is further supported by the observation that the wild-type target exhibited the highest *R*_ct_, indicating the greatest obstruction to electron transfer, whereas the mutant target showed a lower *R*_ct_, suggesting reduced hindrance due to DNA cleavage and subsequent dissociation of the complex. Despite that, these findings indicate that CRISPR/Cas9 systems can discriminate single-base mismatches not only via cleavage activity but also through non-catalytic interactions that influence electrochemical readout. This highlights the potential of leveraging steric hindrance mechanisms in CRISPR-based biosensors for highly specific detection of point mutations.

## Conclusions

In this study, we successfully developed a CRISPR/Cas9-based electrochemical biosensor capable of detecting *katG* gene mutations associated with isoniazid resistance in multidrug-resistant *Mycobacterium tuberculosis* (MDR-TB). By designing a guide RNA with high specificity (on-target score of 61.2 and off-target score of 49.0) targeting the frequently mutated codon 315, we ensured effective recognition and cleavage of the mutant DNA sequence in proximity to the PAM (AGG), a key factor for Cas9 activity. The biosensor employed a gold-modified screen-printed carbon electrode (SPCE/Au), functionalized via streptavidin-biotin interactions to immobilize a ferrocene-labelled DNA probe. Upon hybridization with the target mutant DNA, the Cas9/gRNA complex cleaved the resulting double-stranded DNA, leading to a significant reduction in ferrocene oxidation signal, which was measured electrochemically using SWV. This sensor demonstrated exceptional analytical performance, with a detection limit as low as 7.5530 aM and a linear dynamic range spanning six orders of magnitude (10^1^ to 10^6^ aM). The system effectively discriminated mutant from wild-type sequences; only the mutant target induced Cas9-mediated cleavage and signal reduction. In contrast, despite not being cleaved, the wild-type target also led to signal suppression, likely due to stable binding of the Cas9/gRNA complex and resulting steric hindrance that impeded electron transfer. This observation was supported by EIS data, which showed a significantly higher *R*_ct_ for the wild-type target compared to the mutant target, confirming greater electron transfer obstruction by the non-cleaved Cas9/gRNA/DNA complex. This outcome underscores the sensor’s high specificity and capacity to detect single-nucleotide variations based on catalytic and non-catalytic interactions. These findings highlight the potential of integrating CRISPR/Cas9-based molecular recognition with electrochemical transduction to develop a diagnostic platform for drug-resistant TB, while also addressing a critical diagnostic gap by enabling differentiation between TB and MDR-TB with isoniazid resistance, offering significant implications for improving treatment decisions, particularly in clinical and resource-limited settings. The approach offers advantages in speed, portability, and cost-effectiveness compared to conventional molecular assays. With further validation using clinical samples and potential integration into point-of-care devices, this biosensor could significantly contribute to the early detection and management of MDR-TB.

## Supplementary material

Additional data are available at https://pub.iapchem.org/ojs/index.php/admet/article/view/2766, or from the corresponding author on request.


