# Supplementary material for: CRISPR-Cas9-based electrochemical biosensor for the detection of katG gene mutations in isoniazid-resistant tuberculosis
Source: ADMET DMPK. 2025 Jun 17;13(3):2766. doi: 10.5599/admet.2766 (PMC12205929; doi:10.5599/admet.2766)
Supplement: Supplementary file 1 [file ADMET-13-2766-S1.pdf]

ADMET & DMPK 13(3) (2025) S2767

Open Access : ISSN : 1848-7718

http://www.pub.iapchem.org/ojs/index.php/admet/index

Supplementary material to

CRISPR-Cas9 based electrochemical biosensor for the detection of *katG* gene mutations in isoniazid-resistant tuberculosis

Dika Apriliana Wulandari[1], Muhammad Ihda Hamlu Liwaissunati Zein[2], Salma Nur Zakiyyah[1], Safri Ishmayana[1], Mehmet Ozsoz[1,3], Yeni Wahyuni Hartati[1,4,]* and Irkham[1,4,]*

[1]*Departemen of Chemistry, Faculty of Mathematics and Natural Science, Universitas Padjadjaran, Sumedang 45363, Indonesia*
[2]*Department of Chemistry "Giacomo Ciamician", Alma Mater Studiorum – University of Bologna, Bologna 40126, Italy*
[3]*Department of Biomedical Engineering, Near East University, Mersin 99138, Turkey*
[4]*Study Center of Sensor and Green Chemistry, Faculty of Mathematics and Natural Science, Universitas Padjadjaran, Bandung 40132, Indonesia*

ADMET & DMPK **13(3)** (2025) 2766; https://doi.org/10.5599/admet.2766

Table S1. The pattern of mutations in the rpoB gene among MDR-TB isolates from different geographical areas

Amino acid codon position	Nucleotide changes	Amino acid changes	Geographic region	Ref.
513	CAA → AAA<br>CAA → CCA	Gln → Lys<br>Gln → Pro	Honduras, Uganda	[1]
516	GAC → GTC	Asp → Val	China, Honduras, Iran, Romania, Uganda	
526	CAC → TGC<br>CAC → CTC<br>CAC → CGC<br>CAC → TAC<br>CAC → GAC	His → Cys<br>His → Leu<br>His → Arg<br>His → Tyr<br>His → Asp	Belarus, China, Honduras, Iran, Romania, Uganda	
531	TCG → CAG<br>TCG → TTG<br>TCG → TTC<br>TCG → TGG	Ser → Gln<br>Ser → Leu<br>Ser → Phe<br>Ser → Trp	Belarus, China, Honduras, Iran, Romania, Uganda	
471*	ATG → ATT	Met → Ile	Brazil<br>(*associated with other mutated codons)	[2]
475*	GTG → GGG	Val → Gly		
508*	ACC → CCC	Thr → Pro		
511	CTG → CCG	Leu → Pro		
513	CAA → CCA	Gln → Pro		
516	GAC → GTC	Asp → Val		
522*	TCG → TTC	Ser → Phe		
526	CAC → GAC<br>CAC → TAC<br>CAC → CGC<br>CAC → TGC<br>CAC → AAC	His → Asp<br>His →Tyr<br>His → Arg<br>His → Cys<br>His → Asn		
531	TCG → TTG<br>TCG → TGG	Ser → Leu<br>Ser → Trp		
533	CTG → CCG	Leu → Pro		
539*	TCA → TTC	Ser → Phe		
545	CTG → CCG	Leu → Pro		

Amino acid codon position	Nucleotide changes	Amino acid changes	Geographic region	Ref.
510	CAG → CCG	Gln → Arg	Myanmar	[3]
513	CAA → CCA	Gln → Pro		
516	GAC → GTC<br>GAC → TAC	Asp → Val<br>Asp → Tyr		
517	CAG → CCG	Gln → Pro		
526	CAC → TAC<br>CAC → GAC<br>CAC →CGC<br>CAC → CTC	His → Tyr<br>His → Asp<br>His → Arg<br>His → Leu		
531	TCG → TTG<br>TCG →TGG<br>TCG → TTT	Ser →Leu<br>Ser → Trp<br>Ser → Phe		
533	CTG → CCG	Leu → Pro		
490	CAA → AAA	Gln → Lys	Indonesia (Central Java)	[4]
511	CTG → CGG	Leu → Arg		
513	CAA → GAA<br>CAA → CCA	Gln → Glu<br>Gln → Pro		
516	GAC → GTC	Asp → Val		
526	CAC → CTC<br>CAC → TGC<br>CAC → TCC<br>CAC → TAC	His → Leu<br>His → Cys<br>His → Ser<br>His → Tyr		
531	TCG → TTG<br>TCG → CTG	Ser → Leu<br>Ser → Leu		
535	CCC → CAC	Pro → His		
430	CTC → CCC	Leu → Pro	Vietnam	[5]
432	CAG → AAG	Gln → Lys		
445	CAC → CTC	His → Leu		
446	AAG → CAG	Lys → Gln		
450	TCG → CTG	Ser → Leu		
452	CTC → CCC	Leu → Pro		
505	TTC → CTC	Phe → Leu	Zambia	[6]
513	CAA → GAA	Gln → Glu		
516	GAC → GTC<br>GAC → TAC<br>GAC → TTC	Asp → Val<br>Asp → Tyr<br>Asp → Phe		
522	TCG → GTG	Ser → Val		
526	CAC → TAC<br>CAC → GAC<br>CAC → CTC<br>CAC → TGC	His → Tyr<br>His →Asp<br>His →Leu<br>His →Cys		
531	TCG → TTG<br>TCG → TGG<br>TCG → TTC	Ser → Val<br>Ser → Leu<br>Ser → Phe		
516	GAC → GTC	Asp → Val	Thailand (western region)	[7]
526	CAC → AAC<br>CAC → GAC<br>CAC → TAC	His → Asn<br>His → Asp<br>His → Tyr		
531	TCG → TTG<br>TCG → TGG	Ser → Leu<br>Ser → Trp		
533	CTG → CCC	Leu → Pro		
516	GAC → GTC	Asp → Val	Thailand (northern region)	[8]
522	TCG → TTG	Ser → Val		
526	CAC → CGC<br>CAC → GAC<br>CAC → TAC	His → Arg<br>His → Asp<br>His → Tyr		
531	TCG → TTG	Ser → Leu		

CC BY

Amino acid codon position	Nucleotide changes	Amino acid changes	Geographic region	Ref.
531	TCG → TTG	Ser → Leu	Pakistan	[9]
459	CTG → CGG	Leu →Arg	China (eastern region)	[10]
463	CAG → CAC	Gln → His		
511	CTG → CCG	Leu → Pro		
513	CAA → CCA<br>CAA → CTA	Gln →Pro<br>Gln → Leu		
515	ATG → CTG	Met → Leu		
516	GAC → TAC<br>GAC → GTC	Asp → Tyr<br>Asp → Val		
522	TCG → TTG	Ser → Leu		
526	CAC → TAC<br>CAC → AAC<br>CAC → GAC<br>CAC → CGC<br>CAC → CTC<br>CAC → CAG	His → Tyr<br>His → Asn<br>His → Asp<br>His → Arg<br>His → Leu<br>His → Gln		
531	TCG → TTG	Ser → Leu		
533	CTG → CCC	Leu → Pro		

Table S2. The pattern of mutations in the katG gene among MDR-TB isolates from different geographical areas

Amino acid codon position	Nucleotide changes	Amino acid changes	Geographic region	Ref.
300	TGG → GGG	Trp → Gly	Iran	[1]
315	AGC → ACC<br>AGC → AAC<br>AGC → ATC<br>AGC → AGA	Ser → Thr<br>Ser → Asn<br>Ser → Ile<br>Ser → Arg	Belarus, China, Honduras, Iran, Romania, Uganda	
385	CGG → CCG	Arg → Pro	Iran	
434	CAG → CCG	Gln → Pro	Uganda	
446	AGC → CGC	Ser → Arg	China	
315	AGC → ACC<br>AGC → AAC<br>AGC → ACA<br>AGC →ATC	Ser → Thr<br>Ser → Asn<br>Ser → Thr<br>Ser → Ile	Brazil	[2]
463	CGG → CTG	Arg → Leu		
315	AGC → ACC	Ser → Thr	India, Moldova, Filipina	[11]
285	GGC → CGC	Gly → Arg	Myanmar	[3]
315	AGC → ACC<br>AGC → AAC<br>AGC → ATC	Ser → Thr<br>Ser → Asn<br>Ser → Ile		
191	TGG → CGG	Trp → Arg	Vietnam	[5]
234	GGC → CGC	Gly → Arg		
315	AGC → ACC	Ser → Thr		
315	AGC → ACC<br>AGC → AAC	Ser → Thr<br>Ser → Asn	Zambia	[6]
329	GAC → GAA	Asp → Glu		
315	AGC → ACC	Ser → Thr	Thailand (western region)	[7]
315	AGC → ACC	Ser → Thr	Thailand (northern region)	[8]
299	GGC → AGC	Gly → Ser	Pakistan	[9]
315	AGC → ACC	Ser → Thr		
241	CCC →CGC	Pro → Arg	China (eastern region)	[10]
289	GAG → GGG	Glu →Gly		
391	GCT → GTT	Ala → Val		
315	AGC → ACC	Ser → Thr		

Reference:

[1] S. Rosales-Klintz, P. Jureen, A. Zalutskayae, A. Skrahina, B. Xu, Y. Hu, L. Pineda-Garcia, M.A. Merza, I. Muntean, F. Bwanga, M. Joloba, S.E. Hoffner. Drug resistance-related mutations in multidrug-resistant *Mycobacterium tuberculosis* isolates from diverse geographical regions. *International Journal of Mycobacteriology* **I** (2012) 124-130. http://dx.doi.org/10.1016/j.ijmyco.2012.08.001

[2] F. A. D. de Freitas, V. Bernardo, M. K. Gomgnimbou, C. Sola, H. R. Siqueira, M. A. S. Pereira, F. C. O. Fandinho, H. M. Gomes, M. E. I. Araújo, P.N. Suffys, E. A. Marques, R. M. Albano. Multidrug resistant *Mycobacterium tuberculosis*: A Retrospective *katG* and *rpoB* mutation profile analysis in isolates from a reference center in Brazil. *PLOS One* **9** (2014) e104100. https://doi.org/10.1371/journal.pone.0104100

[3] K. S. Aye, C. Nakajima, T. Yamaguchi, M. M. Win, M. M. Shwe, A. A. Win, T. Lwin, W. W. Nyunt, T. Ti, Y. Suzuki. Genotypic characterization of multi-drug-resistant *Mycobacterium tuberculosis* isolate in Myanmar. *Journal of Infection and Chemotherapy* **22** (2016) 174-179. https://doi.org/10.1016/j.jiac.2015.12.009

[4] M. Erawati, N. S. D. Kusumaningrum, M. Andriany. Mutations in the rpoB gene of multidrug-resistant *Mycobacterium tuberculosis* isolates from Semarang, Indonesia. *International Journal of Molecular and Clinical Microbiology* **7** (2017) 816-823. https://journals.iau.ir/article_537742.html

[5] N. T. L. Hang, M. Hijikata, S. Maeda, P. H. Thuong, J. Ohashi, H. V. Huan, N. P. Hoang, A. Miyabayashi, V. C. Cuong, S. Seto, N. V. Hung, N. Keicho. Whole genome sequencing, analyses of drug resistance-coffering mutations, and correlation with transmission of *Mycobacterium tuberculosis* carrying *katG*-S315T in Hanoi, Vietnam. *Scientific Reports Nature Research* **9** (2019) 15354. https://doi.org/10.1038/s41598-019-51812-7

[6] E. S. Solo, C. Nakajima, T. Kaile, P. Bwalya, G. Mbulo, Y. Fukushima, S. Chila, N. Kapata, Y. Shah, Y. Suzuki. Mutations in rpoB and katG genes and the inhA operon in multidrug-resistant *Mycobacterium tuberculosis* isolates from Zambia. *Journal of Global Antimicrobial Resistance* **22** (2020) 302-307. https://doi.org/10.1016/j.jgar.2020.02.026

[7] K. Suthum, W. Samosornsuk, S. Samosornsuk. Characterization of *katG, rpoB,* and *pncA* in *Mycobacterium tuberculosis* isolates from MDR-TB risk patients in Thailand. *The Journal of Infection in Developing Countries* **14** (2020) 268-276. https://doi.org/10.3855/jidc.11974

[8] U. Anukool, P. Phunpae, C. SItthidet, Tharinjaroen, B. Butr-Indr. S. Saikaew, N. Netirat, S. Intorasoot, V. Suthachai, K. Tragoolpua, A. Chaiprasert. Genotypic distribution and a potential diagnostic assay of multidrug-resistant tuberculosis in Northern Thailand. *Infection and Drug Resistance* **13** (2020) 3375-3382. https://doi.org/10.2147/idr.s263082

[9] A. Aftab, S. Afzal, Z. Qamar, M. Idrees. Early detection of MDR *Mycobacterium tuberculosis* mutations in Pakistan. *Scientific Reports* **11** (2021) 10-14. https://doi.org/10.1038/s41598-021-96116-x

[10] Q. Liu, D. Yang, B. Qiu, L. Martinez, Y. Ji, H. Song, Z. Li, J. Wang. Drug resistance gene mutations and treatment outcomes in MDR-TB: a prospective study in Eastern China. *PLOS Neglected Tropical Diseases* **15** (2021) e0009068. https://doi.org/10.1371/journal.pntd.0009068

[11] J. N. Torres, L. V. Paul, T. C. Rodwell, T. C. Victor, A. M. Amallraja, A. Elghraoui, A. P. Goodmanson, S. M. Ramirez-Busby, A. Chawla, V. Zadorozhny, E. M. Streicher, F. A. Sirgel, D. Catanzaro, C. Rodrigues, M. T. Gler, V. Crudu, A. Catanzaro, F. Valafar. Novel *katG* mutations causing isoniazid resistance in clinical *M. tuberculosis* isolates. *Emerging Microbes and Infectious* **4** (2015) e42. https://doi.org/10.1038/emi.2015.42

CC BY
