# Supplementary material for: CRISPR-Cas9-based electrochemical biosensor for the detection of katG gene mutations in isoniazid-resistant tuberculosis
Source: ADMET DMPK. 2025 Jun 17;13(3):2766. doi: 10.5599/admet.2766 (PMC12205929; doi:10.5599/admet.2766)
Supplement: Supplementary file 2 [file ADMET-13-2766-S1.docx]

*ADMET & DMPK 13(3) (2025) S2767*

*
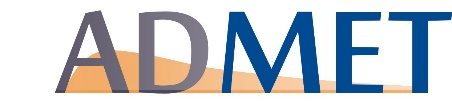
***Open Access : ISSN : 1848-7718**[***http://www.pub.iapchem.org/ojs/index.php/admet/index***](http://www.pub.iapchem.org/ojs/index.php/admet/index)

Supplementary material to

**CRISPR-Cas9 based electrochemical biosensor for the detection of *katG* gene mutations in isoniazid-resistant tuberculosis**

Dika Apriliana Wulandari^1^, Muhammad Ihda Hamlu Liwaissunati Zein^2^,
Salma Nur Zakiyyah^1^, Safri Ishmayana^1^, Mehmet Ozsoz^1,3^, Yeni Wahyuni Hartati^1,4,^* and Irkham^1,4,🟋^

^1^Departemen of Chemistry, Faculty of Mathematics and Natural Science, Universitas Padjadjaran, Sumedang 45363, Indonesia
^2^Department of Chemistry “Giacomo Ciamician”, Alma Mater Studiorum – University of Bologna, Bologna 40126, Italy
^3^Department of Biomedical Engineering, Near East University, Mersin 99138, Turkey
^4^Study Center of Sensor and Green Chemistry, Faculty of Mathematics and Natural Science, Universitas Padjadjaran, Bandung 40132, Indonesia

ADMET & DMPK **13(3)** (2025) 2766; <https://doi.org/10.5599/admet.2766>

**Table S1**. The pattern of mutations in the rpoB gene among MDR-TB isolates from different geographical areas

| Amino acid codon position | Nucleotide changes | Amino acid changes | Geographic region | Ref. |
| --- | --- | --- | --- | --- |
| 513 | CAA 🡪 AAA  CAA 🡪 CCA | Gln 🡪 Lys  Gln 🡪 Pro | Honduras, Uganda | [1] |
| 516 | GAC 🡪 GTC | Asp 🡪 Val | China, Honduras, Iran, Romania, Uganda |  |
| 526 | CAC 🡪 TGC  CAC 🡪 CTC  CAC 🡪 CGC  CAC 🡪 TAC  CAC 🡪 GAC | His 🡪 Cys  His 🡪 Leu  His 🡪 Arg  His 🡪 Tyr  His 🡪 Asp | Belarus, China, Honduras, Iran, Romania, Uganda |  |
| 531 | TCG 🡪 CAG  TCG 🡪 TTG  TCG 🡪 TTC  TCG 🡪 TGG | Ser 🡪 Gln  Ser 🡪 Leu  Ser 🡪 Phe  Ser 🡪 Trp | Belarus, China, Honduras, Iran, Romania, Uganda |  |
| 471* | ATG 🡪 ATT | Met 🡪 Ile | Brazil  (*associated with other mutated codons) | [2] |
| 475* | GTG 🡪 GGG | Val 🡪 Gly |  |  |
| 508* | ACC 🡪 CCC | Thr 🡪 Pro |  |  |
| 511 | CTG 🡪 CCG | Leu 🡪 Pro |  |  |
| 513 | CAA 🡪 CCA | Gln 🡪 Pro |  |  |
| 516 | GAC 🡪 GTC | Asp 🡪 Val |  |  |
| 522* | TCG 🡪 TTC | Ser 🡪 Phe |  |  |
| 526 | CAC 🡪 GAC  CAC 🡪 TAC  CAC 🡪 CGC  CAC 🡪 TGC  CAC 🡪 AAC | His 🡪 Asp  His 🡪Tyr  His 🡪 Arg  His 🡪 Cys  His 🡪 Asn |  |  |
| 531 | TCG 🡪 TTG  TCG 🡪 TGG | Ser 🡪 Leu  Ser 🡪 Trp |  |  |
| 533 | CTG 🡪 CCG | Leu 🡪 Pro |  |  |
| 539* | TCA 🡪 TTC | Ser 🡪 Phe |  |  |
| 545 | CTG 🡪 CCG | Leu 🡪 Pro |  |  |
| 510 | CAG 🡪 CCG | Gln 🡪 Arg | Myanmar | [3] |
| 513 | CAA 🡪 CCA | Gln 🡪 Pro |  |  |
| 516 | GAC 🡪 GTC  GAC 🡪 TAC | Asp 🡪 Val  Asp 🡪 Tyr |  |  |
| 517 | CAG 🡪 CCG | Gln 🡪 Pro |  |  |
| 526 | CAC 🡪 TAC  CAC 🡪 GAC  CAC 🡪CGC  CAC 🡪 CTC | His 🡪 Tyr  His 🡪 Asp  His 🡪 Arg  His 🡪 Leu |  |  |
| 531 | TCG 🡪 TTG  TCG 🡪TGG  TCG 🡪 TTT | Ser 🡪Leu  Ser 🡪 Trp  Ser 🡪 Phe |  |  |
| 533 | CTG 🡪 CCG | Leu 🡪 Pro |  |  |
| 490 | CAA 🡪 AAA | Gln 🡪 Lys | Indonesia (Central Java) | [4] |
| 511 | CTG 🡪 CGG | Leu 🡪 Arg |  |  |
| 513 | CAA 🡪 GAA  CAA 🡪 CCA | Gln 🡪 Glu  Gln 🡪 Pro |  |  |
| 516 | GAC 🡪 GTC | Asp 🡪 Val |  |  |
| 526 | CAC 🡪 CTC  CAC 🡪 TGC  CAC 🡪 TCC  CAC 🡪 TAC | His 🡪 Leu  His 🡪 Cys  His 🡪 Ser  His 🡪 Tyr |  |  |
| 531 | TCG 🡪 TTG  TCG 🡪 CTG | Ser 🡪 Leu  Ser 🡪 Leu |  |  |
| 535 | CCC 🡪 CAC | Pro 🡪 His |  |  |
| 430 | CTC 🡪 CCC | Leu 🡪 Pro | Vietnam | [5] |
| 432 | CAG 🡪 AAG | Gln 🡪 Lys |  |  |
| 445 | CAC 🡪 CTC | His 🡪 Leu |  |  |
| 446 | AAG 🡪 CAG | Lys 🡪 Gln |  |  |
| 450 | TCG 🡪 CTG | Ser 🡪 Leu |  |  |
| 452 | CTC 🡪 CCC | Leu 🡪 Pro |  |  |
| 505 | TTC 🡪 CTC | Phe 🡪 Leu | Zambia | [6] |
| 513 | CAA 🡪 GAA | Gln 🡪 Glu |  |  |
| 516 | GAC 🡪 GTC  GAC 🡪 TAC  GAC 🡪 TTC | Asp 🡪 Val  Asp 🡪 Tyr  Asp 🡪 Phe |  |  |
| 522 | TCG 🡪 GTG | Ser 🡪 Val |  |  |
| 526 | CAC 🡪 TAC  CAC 🡪 GAC  CAC 🡪 CTC  CAC 🡪 TGC | His 🡪 Tyr  His 🡪Asp  His 🡪Leu  His 🡪Cys |  |  |
| 531 | TCG 🡪 TTG  TCG 🡪 TGG  TCG 🡪 TTC | Ser 🡪 Val  Ser 🡪 Leu  Ser 🡪 Phe |  |  |
| 516 | GAC 🡪 GTC | Asp 🡪 Val | Thailand (western region) | [7] |
| 526 | CAC 🡪 AAC  CAC 🡪 GAC  CAC 🡪 TAC | His 🡪 Asn  His 🡪 Asp  His 🡪 Tyr |  |  |
| 531 | TCG 🡪 TTG  TCG 🡪 TGG | Ser 🡪 Leu  Ser 🡪 Trp |  |  |
| 533 | CTG 🡪 CCC | Leu 🡪 Pro |  |  |
| 516 | GAC 🡪 GTC | Asp 🡪 Val | Thailand (northern region) | [8] |
| 522 | TCG 🡪 TTG | Ser 🡪 Val |  |  |
| 526 | CAC 🡪 CGC  CAC 🡪 GAC  CAC 🡪 TAC | His 🡪 Arg  His 🡪 Asp  His 🡪 Tyr |  |  |
| 531 | TCG 🡪 TTG | Ser 🡪 Leu |  |  |
| 531 | TCG 🡪 TTG | Ser 🡪 Leu | Pakistan | [9] |
| 459 | CTG 🡪 CGG | Leu 🡪Arg | China (eastern region) | [10] |
| 463 | CAG 🡪 CAC | Gln 🡪 His |  |  |
| 511 | CTG 🡪 CCG | Leu 🡪 Pro |  |  |
| 513 | CAA 🡪 CCA  CAA 🡪 CTA | Gln 🡪Pro  Gln 🡪 Leu |  |  |
| 515 | ATG 🡪 CTG | Met 🡪 Leu |  |  |
| 516 | GAC 🡪 TAC  GAC 🡪 GTC | Asp 🡪 Tyr  Asp 🡪 Val |  |  |
| 522 | TCG 🡪 TTG | Ser 🡪 Leu |  |  |
| 526 | CAC 🡪 TAC  CAC 🡪 AAC  CAC 🡪 GAC  CAC 🡪 CGC  CAC 🡪 CTC  CAC 🡪 CAG | His 🡪 Tyr  His 🡪 Asn  His 🡪 Asp  His 🡪 Arg  His 🡪 Leu  His 🡪 Gln |  |  |
| 531 | TCG 🡪 TTG | Ser 🡪 Leu |  |  |
| 533 | CTG 🡪 CCC | Leu 🡪 Pro |  |  |

**Table S2.** The pattern of mutations in the katG gene among MDR-TB isolates from different geographical areas

| Amino acid codon position | Nucleotide changes | Amino acid changes | Geographic region | Ref. |
| --- | --- | --- | --- | --- |
| 300 | TGG 🡪 GGG | Trp 🡪 Gly | Iran | [1] |
| 315 | AGC 🡪 ACC  AGC 🡪 AAC  AGC 🡪 ATC  AGC 🡪 AGA | Ser 🡪 Thr  Ser 🡪 Asn  Ser 🡪 Ile  Ser 🡪 Arg | Belarus, China, Honduras, Iran, Romania, Uganda |  |
| 385 | CGG 🡪 CCG | Arg 🡪 Pro | Iran |  |
| 434 | CAG 🡪 CCG | Gln 🡪 Pro | Uganda |  |
| 446 | AGC 🡪 CGC | Ser 🡪 Arg | China |  |
| 315 | AGC 🡪 ACC  AGC 🡪 AAC  AGC 🡪 ACA  AGC 🡪ATC | Ser 🡪 Thr  Ser 🡪 Asn  Ser 🡪 Thr  Ser 🡪 Ile | Brazil | [2] |
| 463 | CGG 🡪 CTG | Arg 🡪 Leu |  |  |
| 315 | AGC 🡪 ACC | Ser 🡪 Thr | India, Moldova, Filipina | [11] |
| 285 | GGC 🡪 CGC | Gly 🡪 Arg | Myanmar | [3] |
| 315 | AGC 🡪 ACC  AGC 🡪 AAC  AGC 🡪 ATC | Ser 🡪 Thr  Ser 🡪 Asn  Ser 🡪 Ile |  |  |
| 191 | TGG 🡪 CGG | Trp 🡪 Arg | Vietnam | [5] |
| 234 | GGC 🡪 CGC | Gly 🡪 Arg |  |  |
| 315 | AGC 🡪 ACC | Ser 🡪 Thr |  |  |
| 315 | AGC 🡪 ACC  AGC 🡪 AAC | Ser 🡪 Thr  Ser 🡪 Asn | Zambia | [6] |
| 329 | GAC 🡪 GAA | Asp 🡪 Glu |  |  |
| 315 | AGC 🡪 ACC | Ser 🡪 Thr | Thailand (western region) | [7] |
| 315 | AGC 🡪 ACC | Ser 🡪 Thr | Thailand (northern region) | [8] |
| 299 | GGC 🡪 AGC | Gly 🡪 Ser | Pakistan | [9] |
| 315 | AGC 🡪 ACC | Ser 🡪 Thr |  |  |
| 241 | CCC 🡪CGC | Pro 🡪 Arg | China (eastern region) | [10] |
| 289 | GAG 🡪 GGG | Glu 🡪Gly |  |  |
| 391 | GCT 🡪 GTT | Ala 🡪 Val |  |  |
| 315 | AGC 🡪 ACC | Ser 🡪 Thr |  |  |

Reference:

1. S. Rosales-Klintz, P. Jureen, A. Zalutskayae, A. Skrahina, B. Xu, Y. Hu, L. Pineda-Garcia, M.A. Merza, I. Muntean, F. Bwanga, M. Joloba, S.E. Hoffner. Drug resistance-related mutations in multidrug-resistant *Mycobacterium tuberculosis* isolates from diverse geographical regions. *International Journal of Mycobacteriology* **I** (2012) 124-130. <http://dx.doi.org/10.1016/j.ijmyco.2012.08.001>
2. F. A. D. de Freitas, V. Bernardo, M. K. Gomgnimbou, C. Sola, H. R. Siqueira, M. A. S. Pereira, F. C. O. Fandinho, H. M. Gomes, M. E. I. Araújo, P.N. Suffys, E. A. Marques, R. M. Albano. Multidrug resistant *Mycobacterium tuberculosis*: A Retrospective *katG* and *rpoB* mutation profile analysis in isolates from a reference center in Brazil. *PLOS One* **9** (2014) e104100. <https://doi.org/10.1371/journal.pone.0104100>
3. K. S. Aye, C. Nakajima, T. Yamaguchi, M. M. Win, M. M. Shwe, A. A. Win, T. Lwin, W. W. Nyunt, T. Ti, Y. Suzuki. Genotypic characterization of multi-drug-resistant *Mycobacterium* *tuberculosis* isolate in Myanmar. *Journal of Infection and Chemotherapy* **22** (2016) 174-179. <https://doi.org/10.1016/j.jiac.2015.12.009>
4. M. Erawati, N. S. D. Kusumaningrum, M. Andriany. Mutations in the rpoB gene of multidrug-resistant *Mycobacterium tuberculosis* isolates from Semarang, Indonesia. *International Journal of Molecular and Clinical Microbiology* **7** (2017) 816-823. <https://journals.iau.ir/article_537742.html>
5. N. T. L. Hang, M. Hijikata, S. Maeda, P. H. Thuong, J. Ohashi, H. V. Huan, N. P. Hoang, A. Miyabayashi, V. C. Cuong, S. Seto, N. V. Hung, N. Keicho. Whole genome sequencing, analyses of drug resistance-coffering mutations, and correlation with transmission of *Mycobacterium tuberculosis* carrying *katG-*S315T in Hanoi, Vietnam. *Scientific Reports Nature Research* **9** (2019) 15354. <https://doi.org/10.1038/s41598-019-51812-7>
6. E. S. Solo, C. Nakajima, T. Kaile, P. Bwalya, G. Mbulo, Y. Fukushima, S. Chila, N. Kapata, Y. Shah, Y. Suzuki. Mutations in rpoB and katG genes and the inhA operon in multidrug-resistant *Mycobacterium tuberculosis* isolates from Zambia. *Journal of Global Antimicrobial Resistance* **22** (2020) 302-307. <https://doi.org/10.1016/j.jgar.2020.02.026>
7. K. Suthum, W. Samosornsuk, S. Samosornsuk. Characterization of *katG, rpoB,* and  *pncA* in *Mycobacterium tuberculosis* isolates from MDR-TB risk patients in Thailand. *The Journal of Infection in Developing Countries* **14** (2020) 268-276. <https://doi.org/10.3855/jidc.11974>
8. U. Anukool, P. Phunpae, C. SItthidet, Tharinjaroen, B. Butr-Indr. S. Saikaew, N. Netirat, S. Intorasoot, V. Suthachai, K. Tragoolpua, A. Chaiprasert. Genotypic distribution and a potential diagnostic assay of multidrug-resistant tuberculosis in Northern Thailand. *Infection and Drug Resistance* **13** (2020) 3375-3382. <https://doi.org/10.2147/idr.s263082>
9. A. Aftab, S. Afzal, Z. Qamar, M. Idrees. Early detection of MDR *Mycobacterium tuberculosis* mutations in Pakistan. *Scientific Reports* **11** (2021) 10-14. <https://doi.org/10.1038/s41598-021-96116-x>
10. Q. Liu, D. Yang, B. Qiu, L. Martinez, Y. Ji, H. Song, Z. Li, J. Wang. Drug resistance gene mutations and treatment outcomes in MDR-TB: a prospective study in Eastern China. *PLOS Neglected Tropical Diseases* **15** (2021) e0009068. <https://doi.org/10.1371/journal.pntd.0009068>
11. J. N. Torres, L. V. Paul, T. C. Rodwell, T. C. Victor, A. M. Amallraja, A. Elghraoui, A. P. Goodmanson, S. M. Ramirez-Busby, A. Chawla, V. Zadorozhny, E. M. Streicher, F. A. Sirgel, D. Catanzaro, C. Rodrigues, M. T. Gler, V. Crudu, A. Catanzaro, F. Valafar. Novel *katG* mutations causing isoniazid resistance in clinical *M. tuberculosis* isolates. *Emerging Microbes and Infectious* **4** (2015) e42. <https://doi.org/10.1038/emi.2015.42>
